# Astrocytes Are Required for Oligodendrocyte Survival and Maintenance of Myelin Compaction and Integrity

**DOI:** 10.3389/fncel.2020.00074

**Published:** 2020-04-02

**Authors:** Reshmi Tognatta, Molly T. Karl, Sharyl L. Fyffe-Maricich, Anastas Popratiloff, Eric D. Garrison, Jessica K. Schenck, Mohammad Abu-Rub, Robert H. Miller

**Affiliations:** ^1^School of Medicine and Health Sciences, George Washington University, Washington, DC, United States; ^2^Gladstone Institute of Neurological Diseases, San Francisco, CA, United States; ^3^Molecular Biology, Ultragenyx, Novato, CA, United States

**Keywords:** astrocytes, myelin maintenance, glutamate, NMDA receptors, electron microscopy

## Abstract

Astrocytes have been implicated in regulating oligodendrocyte development and myelination *in vitro*, although their functions *in vivo* remain less well defined. Using a novel approach to locally ablate GFAP+ astrocytes, we demonstrate that astrocytes are required for normal CNS myelin compaction during development, and for maintaining myelin integrity in the adult. Transient ablation of GFAP+ astrocytes in the mouse spinal cord during the first postnatal week reduced the numbers of mature oligodendrocytes and inhibited myelin formation, while prolonged ablation resulted in myelin that lacked compaction and structural integrity. Ablation of GFAP+ astrocytes in the adult spinal cord resulted in the rapid, local loss of myelin integrity and regional demyelination. The loss of myelin integrity induced by astrocyte ablation was greatly reduced by NMDA receptor antagonists, both *in vitro* and *in vivo*, suggesting that myelin stability was affected by elevation of local glutamate levels following astrocyte ablation. Furthermore, targeted delivery of glutamate into adult spinal cord white matter resulted in reduction of myelin basic protein expression and localized disruption of myelin compaction which was also reduced by NMDA receptor blockade. The pathology induced by localized astrocyte loss and elevated exogenous glutamate, supports the concept that astrocytes are critical for maintenance of myelin integrity in the adult CNS and may be primary targets in the initiation of demyelinating diseases of the CNS, such as Neuromyelitis Optica (NMO).

## Introduction

Astrocytes perform multiple functions in the developing and adult CNS associated with maintaining brain and spinal cord integrity and homeostasis including the release, uptake, and sequestration of neurotransmitters such as glutamate (Abbott et al., 2006; Parpura and Zorec, 2010; Fyffe-Maricich et al., 2013; Lundgaard et al., 2014). Cell culture studies suggest astrocytes also influence the initial generation of oligodendrocytes and their precursors (Noble and Murray, 1984). For example, astrocytes are a major source of growth factors that promote the proliferation, survival and migration of oligodendrocyte precursors (Noble and Murray, 1984; Raff et al., 1988; Richardson et al., 1988). Other astrocytic signals influence CNS myelination through the modulation of OPC proliferation (Richardson et al., 1988), migration (Tsai et al., 2002), differentiation, the induction of myelination (Ishibashi et al., 2006), and modulation of myelin thickness (Fyffe-Maricich et al., 2013).

The mechanisms mediating myelin compaction are complex (Aggarwal et al., 2013; Snaidero et al., 2014). Formation of myelin wraps depends on the disassembly of actin filaments (Zuchero et al., 2015), and subsequent compaction of the cytoplasmic leaflets. This compaction appears to require the expression of myelin basic protein (MBP) since *Shiverer* mice lacking MBP fail to generate significant amounts of compact myelin (Privat et al., 1979; Rosenbluth, 1980) and loss of MBP results in myelin breakdown (Weil et al., 2016). Early studies assumed myelin was highly stable, however, recent work suggests myelin is relatively dynamic and is affected by a variety of stimuli. For example, during motor-skill learning, new myelin is generated in areas of the CNS associated with that task, and suppressing the generation of new oligodendrocytes or myelin impairs this learning capacity (McKenzie et al., 2014; Xiao et al., 2016). Furthermore, sensory enrichment enhances oligodendrocyte integration and myelination in the mature cortex (Hughes et al., 2018).

The role of astrocytes in myelin remodeling and maintenance in the mature CNS is poorly defined (Barnett and Linington, 2013) although several lines of evidence imply a close functional relationship. For example, disruption of junctional contacts between astrocytes and mature oligodendrocytes, results in oligodendrocyte loss and thinner myelin (Menichella et al., 2003; Tress et al., 2012), and deletion of the astrocyte intermediate filament, glial fibrillary acidic protein (GFAP) affects astrocyte integrity and leads to disruption of myelination (Liedtke et al., 1996). More recently, peri-nodal astrocytes in the white matter were proposed to regulate myelin thickness and nodal length by exocytosis of thrombin protease inhibitors (Dutta et al., 2018). Some of the strongest data implicating astrocytes in myelin maintenance comes from studies of the demyelinating disease neuromyelitis optica (NMO). The majority of NMO patients develop autoantibodies to the astrocytic water channel aquaporin-4 (AQP4) (Lennon et al., 2005) that result in functional perturbation and astrocyte loss. In classic NMO the lesions that subsequently develop are characterized by an influx of peripheral immune cells, loss of GFAP immunoreactivity, and demyelination (Roemer et al., 2007). Other NMO lesion types, however, particularly in the spinal cord and brainstem show intense inflammation and loss of AQP4 with relative preservation of myelin and activated GFAP-positive astrocytes, suggesting that demyelination is secondary to astrocyte loss rather than the inflammatory response (Roemer et al., 2007; Watanabe et al., 2007).

Several factors make the analysis of astrocyte functions in the intact CNS challenging. First, astrocytes are highly plastic and can alter their morphology and molecular characteristics in response to different environmental cues (Barnett and Linington, 2013). Second, as a result of their roles in neurotransmitter uptake and modulation of the blood brain barrier, astrocytes are thought to be essential for neuronal function and large-scale perturbation of specific astrocyte subgroups leads to abnormal motor neuron synaptogenesis (Tsai et al., 2012). Previous studies targeting proliferating, reactive astrocytes using the targeted expression of thymidine kinase have provided critical insights into the role of reactive astrocytes in injury responses and repair in the CNS (Voskuhl et al., 2009) but are less informative in the uninjured CNS where cell proliferation is limited.

The current study employs a novel transgenic strategy for selective local elimination of both proliferative and non-proliferative GFAP+ astrocytes through the targeted induction of the apoptotic pathway, allowing for detailed loss-of-function analyses. Here we show that GFAP+ astrocytes are required for normal development and maintenance of myelination. Transient ablation of astrocytes during early postnatal development inhibits myelin formation while extended astrocyte ablation results in reduced, poorly compacted myelin. Furthermore, local targeted ablation of GFAP+ astrocytes in the adult CNS leads to rapid loss of myelin compaction and myelin loss. This pathology is associated with a reduction of MBP expression and is reduced by inhibition of N-methyl-D-aspartate (NMDA) receptors. Furthermore, local delivery of L-glutamic acid into the adult spinal cord white matter resulted in reduction of myelin basic protein immunoreactivity and localized disruption of myelin compaction analogous to that following astrocyte ablation. Blockade of NMDA receptor at the time of L-glutamic acid administration prevented the loss of myelin compaction. These studies provide direct evidence that astrocytes play a pivotal role in myelin compaction and maintenance in the normal CNS and support the notion that they may be an important cellular target in dysmyelinating and demyelinating diseases.

## Materials and Methods

All animal experiments were done in compliance with approved animal policies of the Institutional Animal Care and Use Committees (IACUC) at Case Western Reserve University and The George Washington University School of Medicine and Health Sciences.

### Generation of GFAP-iCP9 Transgenic Mice

The 5265 bp pIRES2 DsRed-Express2 vector (Clontech, 632540) was digested with *Asc*I and *Nhe*I to remove all but the first 7 bp of the CMV promoter to yield the 4673 bp pSFIRESDsRed plasmid. A 1.3 kb fragment containing the inducible Caspase 9 (iCP9) sequence was removed from the pLpMBP(iCP9)MG vector (Caprariello et al., 2012) and inserted into the multiple cloning site (MCS) of pSFIRESDsRed using *Xma*I; yielding a 5973 bp pSFiCP9IRESDsRed plasmid. The mouse GFAP promoter was isolated from the mGFAP-Cre plasmid (provided by Dr. K. McCarthy, University of North Carolina) using *Sac*I and *Bam*HI. The mGFAP promoter was ligated with the *Sac*I linearized pSFiCP9IRESDsRed plasmid followed by blunting of the *Bam*HI site and a second ligation step to generate the final 8286 bp pGFAPiCP9IRESDsRed construct. Isolated clones were restriction mapped to confirm correct orientation and transgenic mice were produced by pronuclear injection of the ∼8 kb cDNA construct (Transgenic and Targeting Core Facility, CWRU).

Thirty-eighth GFAP-iCP9 founders were generated and screened using iCP9 specific PCR primers and positive founders crossed to C57BL/6J animals (Jackson Laboratory). Positive progeny were assessed for DsRed expression in the CNS by immunohistochemistry. Screening yielded two independent founder lines: a high expressing line where ∼90% of GFAP+ astrocytes were positive for DsRed and a low expressing line where ∼20% of GFAP+ astrocytes were positive for DsRed. Both founders were maintained as independent lines. All experimental animals used in this study are from high expressing founder lines and included both male and females. No gender dependent differences to experimental response were noted.

### iCP9 Induction and Glutamate Delivery

iCP9 was activated by delivery of a small molecular chemical inducer of dimerization (CID) (Clontech, 635058) into the intrathecal space or directly into the CNS parenchyma of GFAP-iCP9 and wildtype (WT) littermate controls. In neonatal animals, CID was administered intrathecally on postnatal days 4, 5, and 6 as described previously (Caprariello et al., 2015). Briefly, a 30-gauge Hamilton syringe needle was inserted into the spinal column at a depth of 2 mm and withdrawal of clear CSF indicated intrathecal targeting. Two μmicroliters of CID (2.5 mM) was slowly dispensed. In adult animals, 2 μl CID (2.5 mM) was injected into the ventral funiculus. Briefly, the dorsal spinal cord surface was exposed between three segments of the thoracic column (T11/13). A pulled glass needle was inserted until it hit the bone, withdrawn 0.3–0.4 mm and 2 μl of CID (2.5 mM) slowly delivered. Similarly, subsequent to craniotomies, 2 μl of CID (2.5 mM) was injected into the corpus callosum at the following stereotaxic co-ordinates in relation to bregma: +1 mm anterior, +0.2 mm left lateral, and 1.8 mm deep. Direct delivery of glutamate and its inhibitors into ventral white matter of the spinal cord, was accomplished using a similar approach to CID injection, with delivery of 2 μl of 100 μM glutamate over a 5 min interval. For co-application experiments, NMDA receptor antagonists, MK801 (25 μM; Abcam, Ab120027) and QNZ46 (20 μM; Tocris, 4801), were delivered simultaneously with glutamate. Controls for the glutamate injection experiments included sham operated and vehicle only delivery. At the indicated times, animals were terminally anesthetized with avertin, perfused with 4% PFA and dissected tissue (brain, spinal cord) was post-fixed at 4°C. The samples were equilibrated in 20% sucrose and 20 μm cryosections prepared on a cryostat microtome (Leica).

### Cell Cultures

Enriched populations of OPCs were isolated from the P2 forebrain of iCP9 transgenic animals, by immunopanning with the monoclonal antibody A2B5 (Robinson and Miller, 1996) and grown in serum-free media: DMEM/F12 (1:1) (Gibco 11320-033) supplemented with N-2 Max Supplement (R&D AR009), B-27 Supplement without vitamin A (Gibco 12587-010), and Glutamax (Gibco 35050-061). In some studies, bFGF (20 ng/ml, R&D 233-FB-025), PDGF (20 ng/ml, R&D 221-AA-050), Sonic Hedgehog (200 ng/ml, R&D 1845-SH-025) and Noggin (100 ng/ml, R&D 3344-NG-050) were added. Cells were plated on poly-l-lysine (PLL)-coated coverslips, in base media with 10% FBS and PDGF, and after 12 h switched to serum-free media. Cultures were treated with 20 μM CID and assayed at 4 and 24 h. Cells were labeled with antibodies to GFAP to identify astrocytes and a combination of monoclonal antibodies A2B5, O4, and CC1 to identify oligodendrocyte lineage cells.

### Mouse Cerebellar Slice Cultures

Three hundred μmicrometers thick cerebellar slices were prepared from P10 mouse cerebellum using a Leica vibratome (Leica, VT1000S, Germany) and immediately placed into cell-culture inserts (0.4 μm, Millicell-CM, Millipore). Slices were grown in medium containing 50% basal Eagle medium (Gibco, 21010-046), 15% heat-inactivated horse serum (Gibco, 16050-114), 25% Hank’s solution (Sigma, H4641), 0.5% glucose (Sigma, G8769), 1% glutamax, and penicillin-streptomycin (Cellgro, 30-001-CI). After 4DIV, slices were treated with either 100 μM CID or 100 μM L-glutamic acid (Sigma) for 24 h. The slices were fixed with 4% PFA for 10 min and delipidated in ice-cold methanol for 10 min. Prior to antibody staining, samples were blocked in 0.1% Triton, 15% NGS, 10% BSA for 1.5 h followed by 0.1% saponin, 5% NGS, 5% BSA for 3 h at room temp. Slices were then incubated with primary and secondary antibodies as described below. To quantify the release of MBP, conditioned media was collected 24 h after glutamate treatment in serum free media and assayed for MBP expression by ELISA (Cusabio Biotech, CSB-E08285m). The samples were prepared and analyzed in triplicate.

### Immunohistochemistry and Cell Quantification

For immunofluorescent antibody staining samples were incubated for 1 h at room temperature in 5% NGS, 0.3% Triton in 1x PBS, followed by primary antibodies overnight at 4°C, and secondary antibodies for 1 h at room temperature. Antigen retrieval was performed using Reveal Decloaker solution (Biocare Medical, RV1000M) for Caspase-3 and mouse anti-GFAP double labeling. Dilutions and sources of primary antibodies are shown in Table 1. Labeling with monoclonal antibodies A2B5, O4, and O1 was performed on live cells. Sections were mounted in Vectashield with DAPI (Vector Laboratories, H-1200). Images were acquired using a Leica DM5500B upright fluorescence microscope. Field of view for cell counts was 0.3 mm^2^. Raw images were post-processed in Photoshop (Adobe systems), as follows: maximum brightness was rescaled to ∼80% and a gamma correction factor of ∼1–1.2 introduced.

**TABLE 1:** Clone, source, and dilution of antibodies.

**Antibodies**	**Source**	**Cat no.**	**Clone**	**Lot no.**	**Dilution**
**PRIMARY**					
Mouse anti-GFAP	Millipore	MAB360	GA5	2739178	1:250
Rabbit anti-GFAP	DakoCytomation	Z0334		2002331	1:250
Mouse anti-NeuN	Millipore (Chemicon)	MAB377	A60	2500605	1:250
Rabbit anti-DsRed	Clontech	632496	Rb ID:1510004	1509043	1:100
Rabbit anti-Olig2	Millipore	AB9610		2701583	1:250
Rabbit anti-Caspase 3	CellSignaling Technology	9661S	ASP175	43-06/2014	1:100
Mouse anti-BrdU	Roche	11170376001	BMC9318	10310200	1:100
Mouse anti-APC (CC1 clone)	Millipore (Calbiochem)	OP80	CC1	2760465	1:400
Rat anti-MBP	Abcam	Ab7349		GR188102-10	1:100
Rabbit anti-PLP	Abcam	Ab28486		GR213628-1	1:200
Rabbit anti-Iba1	Wako Chemicals	01919741		LKN4881	1:250
Monoclonal mouse anti-A2B5	Hybridoma (gift)				1:2
Monoclonal mouse anti-O4/O1	Hybridoma (gift)				1:4
**SECONDARY**					
Goat anti-mouse Alexa 594	Invitrogen	A11032	–	1741781	1:500
Goat anti-mouse Alexa 488	Invitrogen	A11029	–	1789729	1:500
Goat anti-rabbit Alexa 594	Invitrogen	A11012	–	1704538	1:500
Goat anti-rat Alexa 594	Invitrogen	A11007	–	1697164	1:500
Goat anti-mouse IgM Alexa 488	Invitrogen	A21042	–	1567215	1:500

### FIBSEM Microscopy Analysis

For ultrastructural analyses, animals were anesthetized and perfused with 2% glutaraldehyde/4% paraformaldehyde in 0.1 M sodium cacodylate buffer, pH 7.4. 400 μm sections were post-fixed in 1% OsO_4_, dehydrated through a series of graded ethanol, incubated in saturated uranyl acetate and embedded in a Poly/Bed812 Resin. One μmicrometer coronal sections were stained with toluidine blue and areas for ultrathin sections selected for EM. Ultrathin spinal cord sections (120 nm) were placed on silicon wafers and SEM images generated with a FEI Helios FIBSEM. High-resolution imaging was done using FEI MAPS software interacting with the scanning stage on the Helios. Tile-scan area was defined based on features such as axons and cell bodies clearly detectable at low magnification. High-resolution images were taken using the following parameters: magnification – 80,000×; accelerating voltage – 2 kV and exported as ^∗^. RAW data files to Photoshop.

### Statistical Analyses

Cell counts were performed from defined regions in at least 2–3 brain sections from 3–4 animals for each time point, from both the WT and GFAP-iCP9 groups. Axon counts were performed from at least two areas of the spinal cord and included at least two animals per group. Unless otherwise specified, data is expressed as mean ± SEM. Data statistical analysis was performed using Student’s unpaired *t*-tests and Two-way Anova with Bonferroni *post-hoc* test where applicable. *P*< 0.05 were considered statistically significant.

## Results

### Local Induction of GFAP+ Astrocyte Ablation Results in Loss of Astrocytes and Oligodendrocyte Lineage Cells

**FIGURE 1:**
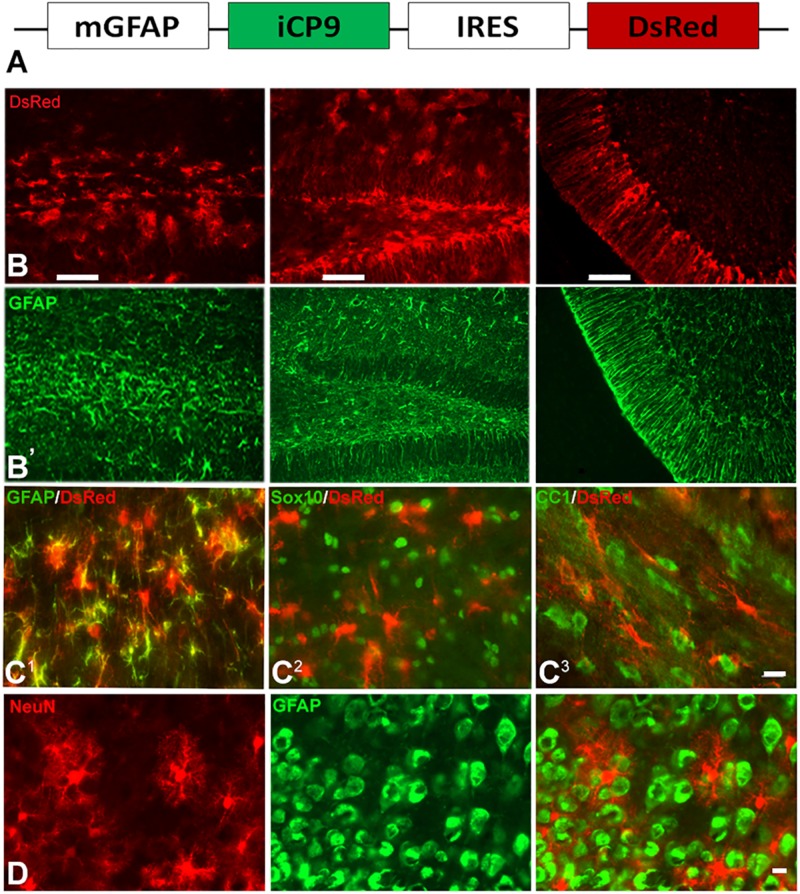
GFAP promoter-driven inducible caspase 9 selectively targets astrocytes. **(A)** Diagram of the construct used to generate the GFAP-iCP9 transgenic animals. The mouse GFAP promoter (mGFAP) drives expression of iCP9 and an IRES-linked DsRed reporter. **(B)** DsRed expression was targeted to astrocytes throughout the CNS. Consecutive sections through the corpus callosum, hippocampus and cerebellum of GFAP-iCP9 animals labeled with antibodies to DsRed (top row, **B**) and GFAP (bottom row, **B’**). The patterns of labeling were highly aligned. **(C)** Coronal sections through the corpus callosum of GFAP-iCP9 mice co-labeled with antibodies to DsRed (red) and GFAP **(C^1^)**, Sox10 **(C^2^)**, and CC1 **(C^3^)** in green. The majority of GFAP+ cells expressed DsRed. By contrast, no Sox10 or CC1+ cells expressed detectable DsRed. **(D)** Coronal section through the frontal cortex of GFAP-iCP9 animals double labeled with antibodies to DsRed (red) and NeuN (green). No NeuN+ cells expressed detectable DsRed. Scale bars: **(B)** 100 μm; **(C)** 20 μm; **(D)** 12.5 μm.

To assay the effects of local depletion of GFAP+ astrocytes, transgenic animals were generated in which an inducible caspase 9 (iCP9) construct (Caprariello et al., 2012) was expressed under the transcriptional control of a mouse glial fibrillary acidic protein (mGFAP) promoter (GFAP-iCP9) (Casper and McCarthy, 2006), resulting in astrocyte-specific expression of the iCP9 construct. Local administration of CID in GFAP-iCP9 mice promotes dimerization of the iCP9 construct, stimulating the physiological apoptotic pathway in targeted cells resulting in cell death. The iCP9 gene was linked by an internal ribosome entry site (IRES) sequence to a DsRed reporter, thereby identifying DsRed-expressing astrocytes in the CNS as susceptible to CID-induced apoptosis (Figure 1A). In the highest expressing line used in this study DsRed had an expression pattern similar to GFAP+ astrocytes in all regions of the CNS (Figure 1B) and 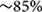 of GFAP+ astrocytes expressed detectable levels of DsRed (208/239 cells/unit area in corpus callosum, *n* = 3). By contrast, no Sox10+ cells (0/496 cells/unit area) or CC1+ oligodendrocytes (0/279 cells/unit area, *n* = 3) expressed detectable DsRed (Figure 1C). In the cortical gray matter, no NeuN+ neurons (*n* = 3) expressed detectable DsRed (Figure 1D) indicating the mGFAP promoter selectively targets iCP9 to GFAP+ cells that morphologically resemble astrocytes. *In vivo*, CID has an active half-life on the order of 5 h, and a single injection of CID (2 μL of 2.5 mM) into the CSF through the cisterna magna of adult animals resulted in the death of 100% of GFAP-iCP9 transgenic and 0% WT animals within 24–36 h (data not shown), confirming that widespread ablation of GFAP+ cells in the adult CNS is lethal. The GFAP-iCP9 transgenic animals were born at the anticipated ratio, had a normal life span and normal CNS cytoarchitecture. There was no significant difference in the number of GFAP+, CC1+ cells and/or axons in the brain and spinal cords of adult (P40) GFAP-iCP9 transgenic mice and WT littermate controls.

**FIGURE 2:**
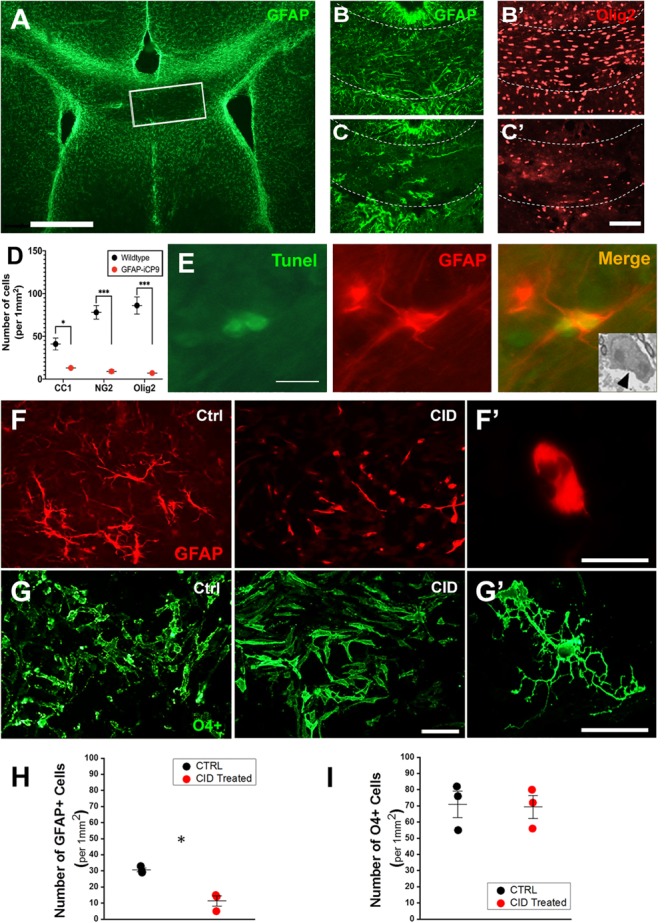
Oligodendrocyte lineage cells are not directly affected by CID treatment but lost following astrocyte ablation *in vivo*. **(A)** Local delivery of CID to the corpus callosum (outlined box) of P30 GFAP-iCP9 animals results in highly localized loss of GFAP+ astrocytes (green) after 24 h. **(B,C)** Representative images from WT **(B)** or GFAP-iCP9 **(C)** animals 12 h after local delivery of CID to the corpus callosum labeled for astrocytes (GFAP, green) and oligodendrocyte lineage cells (Olig2, red). The loss of GFAP+ astrocytes was accompanied with local reductions in Olig2+ oligodendrocyte lineage cells. **(D)** Graph representing numbers of CC1+, NG2+, and Olig2+ cells per unit area in wildtype and GFAP-iCP9 treated mice. **(E)** 12 h after CID delivery, Tunnel+ (green)/GFAP+ (red) cells were present in (the injected region. The inset represents an EM image showing characteristic apoptotic morphology in a likely astrocyte 12 h after CID delivery (black arrow). **(F)** In A2B5 enriched cortical cultures from GFAP-iCP9 animals exposed to CID, astrocytes (labeled with GFAP; red) underwent apoptosis as denoted by the presence of fragmented thinner cellular processes. **(F’)** Higher magnification image of a GFAP+ cell after exposure to CID showing an atrophic astrocyte devoid of cellular processes. **(G)** Oligodendrocyte lineage cells labeled with a mixture of A2B5, O4, and O1 antibodies (green), were unaffected in number **(G)** and morphology **(G’)** by exposure to CID. **(H)** Graph representing number of GFAP+ cells per unit area showing significant loss after CID delivery. **(I)** In contrast, no change in the number of oligodendrocyte lineage cells was seen after CID delivery *in vitro*. Graphs in **(D,H,I)** represent mean number of cells ± SEM. Two-way ANOVA with Sidak’s multiple comparison tests in **(D)**, and student’s unpaired *t*-test in **(H,I)**. * indicates *p* < 0.05, *** indicates *p* < 0.0001. Scale bars: **(A)** 500 μm; **(B,C)** 100 μm; **(D)** 10 μm; **(F,G)** 20 μm.)

The specificity of astrocyte ablation in the GFAP-iCP9 model was confirmed using a combination of *in vivo* and *in vitro* approaches. CID injection (2 μL of 2.5 mM) into the center of the corpus callosum in adult GFAP-iCP9 animals (P30-P50) resulted in localized loss of GFAP+ cells (Figures 2A,C) while CID injections into WT littermate controls had no detectable effects on cell number or morphology (Figure 2B). Astrocyte ablation was also associated with a rapid reduction in the density of CC1+, NG2+, and Olig2+ oligodendrocyte lineage cells (Figures 2C’,D) after 24 h. Somewhat unexpectedly, no disruption of the blood brain barrier (BBB) was seen in the first 24 h following CID injection, demonstrated by the lack of FITC-dextran (40 kDa) extravasation into the surrounding brain parenchyma (data not shown). Astrocyte loss was mediated through apoptosis as indicated by increased TUNEL immunoreactivity in GFAP+ cells (Figure 2E), and the presence of apoptotic morphology of astrocytes (Figure 2E inset).

*In vitro*, addition of CID (20 μM) to cultures of neonatal cortical cells from GFAP-iCP9 animals resulted in detectable astrocyte loss after 4 h followed by extensive loss of all cell types including oligodendrocytes within 24 h. The absence of DsRed and iCP9 expression in OPCs and oligodendrocytes suggested their loss following CID addition was indirect and their survival *in vitro* was dependent on viable astrocytes. To directly assess the effects of CID treatment on oligodendrocyte lineage cells, an enriched population of A2B5+ OPCs were isolated from GFAP-iCP9 animals and grown for 3 days in serum-free conditions prior to CID treatment with added growth factors to replace astrocyte derived survival cues. In such cultures a low proportion of cells were GFAP+ and these underwent apoptosis within 12 h of CID treatment (Figures 2F,H), while there was no significant effect on the number of O4/CC1+ cells (Figures 2G,I) indicating that CID has no direct effect on GFAP-iCP9 derived oligodendrocyte lineage cells. The loss of oligodendrocyte lineage cells *in vivo* is likely a secondary effect following astrocyte ablation since oligodendrocyte lineage cells in GFAP-iCP9 animals lack DsRed expression and their survival in purified cultures was unaffected by CID exposure suggesting the maintenance of oligodendrocyte lineage cells is dependent on GFAP+ astrocytes.

### GFAP+ Astrocytes Are Required for Normal Myelination During Early Postnatal Development

**FIGURE 3:**
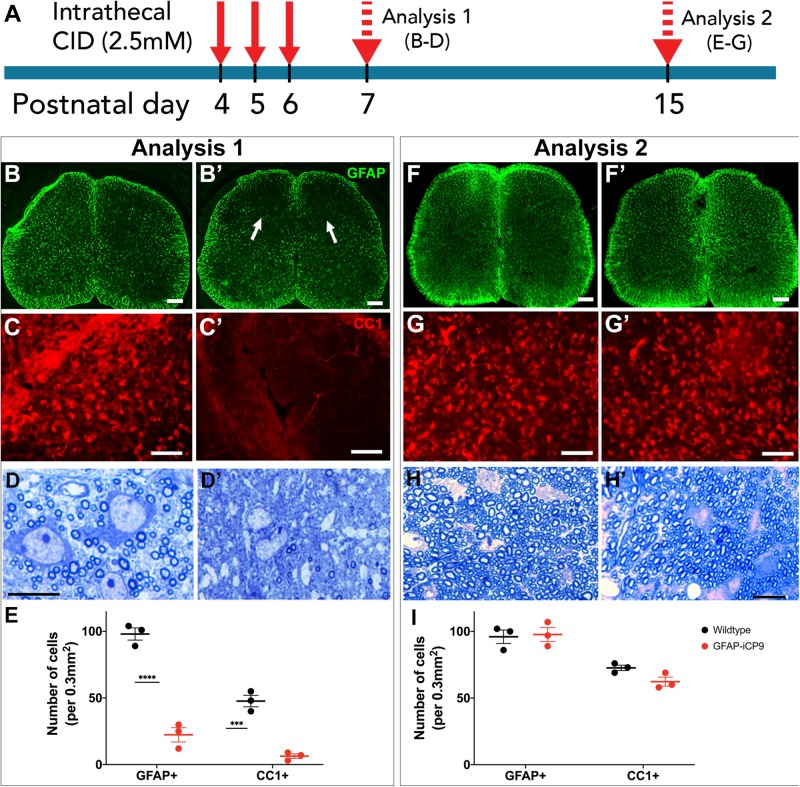
Astrocyte loss compromises spinal cord myelination. **(A)** Experimental design. Daily intrathecal injections of CID to T11/13 segments of spinal cord were delivered on postnatal days 4,5,6 and the animals analyzed on postnatal days 7 (analysis 1) and 15 (analysis 2). **(B)** On p7, compared to controls **(B)** CID treatment significantly reduced the expression of GFAP **(B’)**. **(C)** Compared to controls **(C)**, CID treatment also significantly reduced the number of CC1+ oligodendrocytes **(C’)**. **(D)** Toluidine blue stained plastic 1μm thick sections confirmed a reduction of myelination compared to controls **(D)** following CID induced loss of astrocytes at p7 **(D’)**. **(E)** Quantification of the relative density of GFAP+ and CCI+ cells in WT and CID treated GFAP- iCP9 spinal cords at P7. **(F,G)** The cell loss was transient and at P15 the number of GFAP+ astrocytes and CC1+ oligodendrocytes was essentially indistinguishable between control **(F,G)** and CID treated **(F’,G’)** regions of GFAP-iCP9 tissue. **(H)** Myelination recovers to control levels **(H)** at P15 **(H’)** following cessation of CID treatment at P6. **(I)** Quantification of the relative density of GFAP+ and CCI+ cells in WT and GFAP- iCP9 spinal cords at P15. Data represent mean number of cells ± SEM. Two-way ANOVA with Sidak’s multiple comparisons test. **** indicates *p* < 0.0001. Scale bars: **(B,F)** 100 μm; **(C,G)** 85 μm; **(D,H)** 20 μm.

To assess the effects of astrocyte ablation on the development of myelination, CID was administered intrathecally at the level of T11/T13 segments of the spinal cord of GFAP-iCP9 and WT littermate controls. Injections were performed on postnatal days 4, 5, and 6, and the cellular composition of the dorsal white matter assayed at P7 (Figure 3A). Although fragments of GFAP+ processes persisted in the affected region, administration of CID resulted in an 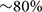 reduction in the number of astrocytes in GFAP-iCP9 but not WT control animals (Figures 3B,E). Astrocyte loss was also accompanied by lower numbers of CC1+ oligodendrocytes (Figures 3C,E), and less myelinated axons (Figure 3D). No change in the density of astrocytes, oligodendrocytes or myelin was detected in regions of the spinal cord distal from the site of CID injection (not shown). The loss of astrocytes, associated oligodendrocytes and myelin was, however, transient: GFAP-iCP9 animals that received CID between P4-6 and were allowed to recover until P15, contained comparable numbers of GFAP+ astrocytes to their WT counterparts (WT 96 ± 5.0 cells/unit area; GFAP-iCP9 97.6 ± 5.2 cells/unit area; *p* > 0.05) (Figures 3F,I) as well as CC1+ oligodendrocytes (WT 72.6 ± 2.0 cells/unit area; GFAP-iCP9 62.3 ± 3.3 cells/unit area; *p* > 0.05) (Figures 3G,I) and myelinated axons (Figure 3H) in CID treated regions.

**FIGURE 4:**
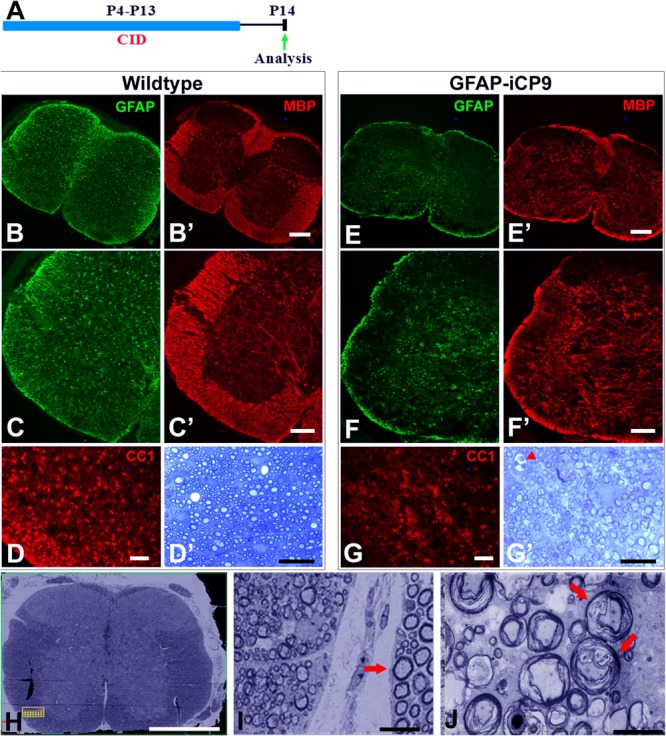
Extended intervals of astrocyte loss compromises myelin integrity. **(A)** Experimental design. Intrathecal injections of CID were done every other day between P4 and P13 and animals analyzed on P14. **(B–D)** In WT spinal cord following CID treatment GFAP+ astrocytes (green) were abundant in gray and white matter **(B,C)** and MBP expression (red) was normal with intense expression in white matter and significantly lower expression in gray matter **(B’,C’)**. The density of CC1+ cells **(D)** and levels of myelination as assessed quantitatively with toluidine blue on 1μm sections **(D’)** were normal. By contrast, in GFAP-iCP9 spinal cords following CID treatment GFAP+ astrocytes **(E,F)** and levels of MBP expression **(E’,F’)** were significantly reduced in white matter such that there was no obvious distinction between expression in white and gray matter. **(G)** The relative number of CC1+ cells was reduced in white matter of CID treated GFAP-iCP9 spinal cords **(G)** compared to WT controls, and myelin appeared disrupted **(G’)**. **(H,I)** In GFAP-iCP9 spinal cords CID injection resulted in disruption of myelin integrity and decompaction. **(H)** Low magnification FIBSEM image confirms lack of contrast between gray and white matter in CID treated segments. **(I)** The decompaction of the myelin following astrocyte loss was specific to the CNS and adjacent PNS myelin (indicated by arrow) was unaffected. **(J)** Higher magnification showing extent of loss of myelin compaction (red arrows). Scale bars: **(B,E)** 200 μm, **(C,F)** 100 μm; **(D,G)** 50 μm; **(D’,G’)** 20 μm; **(H)** 400 μm; **(I)** 10 μm; **(J)** 5 μm.

To determine if prolonged loss of GFAP+ astrocytes compromised myelin formation, CID was administered intrathecally every other day from P4 to P13; an interval largely spanning the period of spinal cord myelination. Tissue was analyzed at P14 (Figure 4A). Glial populations in the WT spinal cords were unaffected by CID treatment and GFAP+ astrocytes were uniformly distributed in gray matter and radially oriented in white matter. The expression of MBP was far more pronounced in white than gray matter and CC1+ oligodendrocytes were abundant in white matter (Figures 4B–D). By contrast, in CID treated regions of GFAP-iCP9 animals, GFAP expression was greatly reduced, and radially oriented GFAP+ processes in the white matter were largely absent or fragmented. The relative distribution of MBP immunoreactivity was also altered. The expression of MBP in white matter was greatly reduced to levels equivalent to or lower than those in gray matter. Although not totally absent, the number of CC1+ oligodendrocytes was also substantially reduced in the CID treated regions compared to controls (Figures 4E–G). The reduction in MBP immunoreactivity in CID treated white matter regions was correlated with a lack of myelin compaction. In sections through CID treated regions, myelin compaction was severely compromised around the majority of large diameter axons (Figures 4H–J). There was also little evidence of a large inflammatory response around blood vessels in CID treated regions (Figure 4G’ arrowhead), suggesting the failure of myelin compaction is the result of astrocyte loss rather than a secondary inflammatory response. The failure of myelin compaction associated with astrocyte loss was localized and specific to the CNS. Peripheral myelin adjacent to affected spinal cord showed normal compaction (Figure 4I, arrow). These data suggest that GFAP+ astrocytes are important for the normal formation of compact myelin *in vivo*.

### Astrocyte Ablation in Adult Spinal Cord White Matter Results in Rapid Loss of Myelin Integrity

**FIGURE 5:**
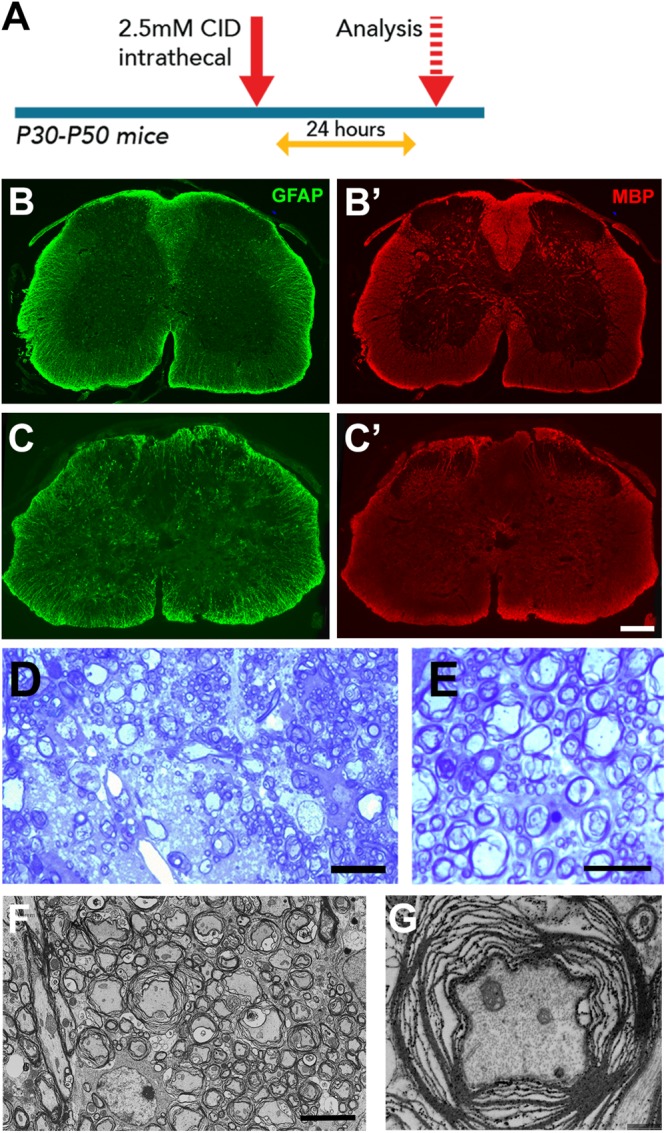
Astrocyte loss *in vivo* resulted in local disruption of myelin integrity. **(A)** Experimental design. **(B,C)** Compared to control segments **(B,B’)**, CID treated segments of GFAP-iCP9 animals had a reduction in GFAP+ cells **(C)** and reduction in MBP expression **(C’)**. **(D,E)** Representative toluidine blue stained images of 1μm spinal cord sections from adult GFAP-iCP9 mice after intrathecal injection of CID. Local astrocyte loss resulted in loss of myelin **(D)** and myelin decompaction **(E)**. **(F,G)** Ultrastructural analysis confirms loss of myelin integrity while axons remain morphologically relatively normal **(G)**. Scale Bars: **(D)** 20 μm; **(E)** 5 μm; **(F)** 5 μm; **(G)** 1 μm; **(B,C)** 200 μm.

Astrocyte injury has been implicated in demyelinating conditions such as NMO, however, it is unclear if the myelin pathology directly reflects astrocyte loss or is secondary to inflammatory responses. To assess whether GFAP+ astrocytes were required for the maintenance of myelin integrity in the mature spinal cord, CID was delivered intrathecally at T11/13 to adult GFAP-iCP9 and WT control animals, and the tissue analyzed after 24 h (Figure 5A). Delivery of CID to WT animals had no apparent effect on gray and white matter patterning or on the expression of GFAP or MBP immunoreactivity (Figures 5B,B’). In GFAP-iCP9 animals there was reduced density of GFAP+ astrocytes and disruption of MBP immunoreactivity in treated regions (Figures 5C,C’). Histological analyses revealed disruption of myelin compaction in CID treated GFAP-iCP9 but not WT animals. Toluidine blue staining showed a reduction in myelin content in affected regions (Figures 5D,E), and there was evidence of myelin perturbation by EM (Figures 5F,G). The loss of astrocytes was more complete in regions close to the site of CID delivery and resulted in local patchy demyelination (Figure 5D), while in adjacent regions there was loss of myelin compaction but no frank demyelination (Figures 5D–F). Although the myelin was severely disrupted, axonal morphology appeared relatively normal (Figure 5G). Spinal cord regions in GFAP-iCP9 animals distal from the injection site had normal glial cytoarchitecture and myelin compaction, demonstrating a localized effect of targeted astrocyte ablation. The rapid disruption of myelin compaction following astrocyte loss is consistent with the hypothesis that in the adult spinal cord astrocytes are critical for maintenance of myelin compaction.

### Myelin Disruption Follows Astrocyte Loss in Slice Cultures

**FIGURE 6:**
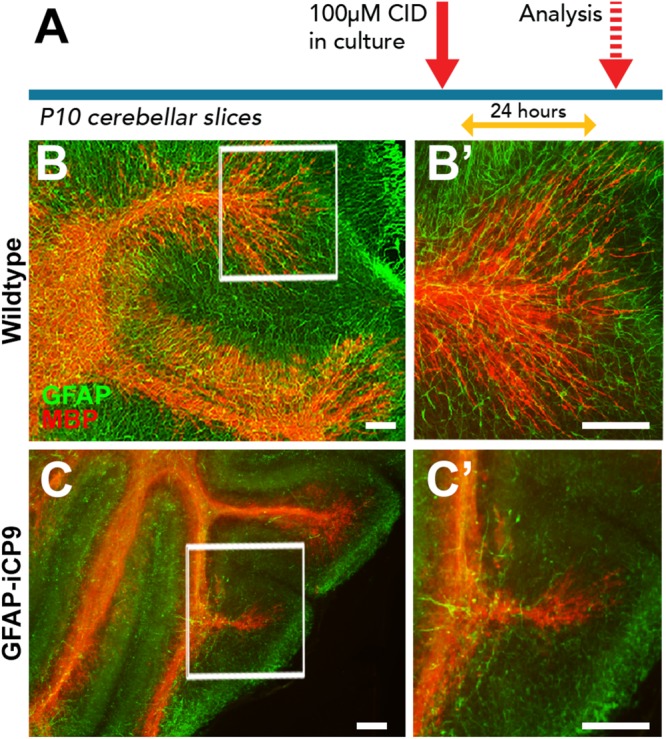
Astrocyte loss in *ex vivo* cerebellar slice cultures resulted in disruption of myelin integrity. **(A)** Experimental design. **(B,C)** Representative images of cerebellar slice cultures co-immunolabeled for MBP (red) and GFAP (green). Compared to controls **(B,B’)** the organization of myelin in GFAP-iCP9 derived cerebellar slice cultures is compromised following CID induced astrocyte loss **(C,C’)**. Scale bars 100 μm.

The perturbation of myelin structure following astrocyte loss *in vivo* may reflect either a direct loss of astrocyte support or a secondary consequence of vascular disruption and changes in the blood brain barrier. To distinguish between these possibilities, myelinating slice cultures of P9 cerebellum from GFAP-iCP9 and WT animals were grown on tissue culture inserts for 4 days and treated with 100 μM CID or vehicle for 24 h (Figure 6A). In control slices treated with CID, or vehicle-treated GFAP-iCP9 slices, a well-organized array of GFAP+ cells supported extensive myelination characterized by extensive linear MBP+ processes (Figures 6B,B’). By contrast, GFAP-iCP9 slices treated with CID demonstrated a reduction in GFAP expression and perturbation of astrocyte patterning, which was associated with a reduction in MBP immunoreactivity (Figure 6C) and a fragmentation of MBP+ myelinated processes (Figure 6C’), consistent with a direct requirement for GFAP+ astrocytes to maintain normal myelin compaction in the CNS.

### Local Increases in Glutamate Mediate Loss of Myelin Compaction Following Astrocyte Death

**FIGURE 7:**
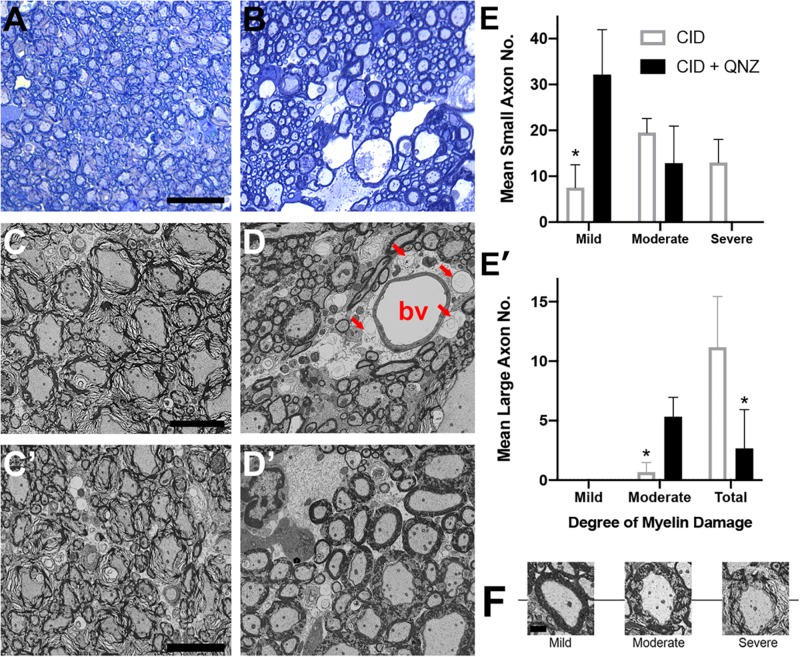
The effects of astrocyte ablation on loss of myelin integrity are reduced by inhibition of NMDA receptors. **(A–D)** Segments of the GFAP-iCP9 spinal cords affected by local delivery of CID into the ventral white matter show severe disruption of myelin sheath integrity after 24–48 h as demonstrated by Toluidine blue light **(A)** and EM **(C)**. By contrast co- injection of QNZ46 provides protection of myelin sheath integrity in the presence of astrocyte loss **(B,D)**. Note that in **(D)** although the astrocytes (denoted by arrows) surrounding the blood vessel (denoted by *bv*) have been lost in QNZ46 co-injected animals the adjacent myelin sheaths are relatively intact. Large diameter myelin sheaths are less protected than small and medium diameter sheaths **(D’)**. **(E)** Quantitation of the relative levels of perturbed myelin sheaths in the presence of CID and CID + QNZ46, from axons of different diameters: Small **(E)**, and large **(E’)**. **(F)** Representative examples of mild, moderate and severely affected axons. Graphs represent mean axon number ± SD. Two-way ANOVA with Sidak’s multiple comparison tests. * indicates *p* < 0.001. Scale bars: **(A,B)** 25 μm; **(C,D)** 5 μm.

Astrocytes are important in maintaining glutamate homeostasis in the CNS through the functions of glutamate transporters (Rothstein et al., 1996), and depletion of GFAP+ astrocytes has been shown to significantly lower the expression of glutamate transporters (Cui et al., 2001). Altered glutamate homeostasis has also been implicated in demyelinating diseases of the CNS. In MS, for example, levels of glutamate increase to excitotoxic levels, where they correlate with the number of active plaques and overall disease burden (Sarchielli et al., 2003; Tisell et al., 2013; Azevedo et al., 2014). Recent studies suggest that elevation of extracellular glutamate affects myelin organization, an effect that is mediated by NMDA receptors in non-MS tissue (Fu et al., 2009; Doyle et al., 2018). To determine whether the loss of myelin compaction seen following astrocyte ablation was mediated by glutamate signaling through NMDA receptors, the NMDA receptor antagonists MK801 and QNZ46 (Doyle et al., 2018) were delivered simultaneously with CID into GFAP-iCP9 spinal cords and the effects of astrocyte loss on myelin compaction assayed. Following either delivery of CID alone or co-delivery of CID and either NMDA receptor antagonist to GFAP-iCP9 spinal cords, equivalent levels of astrocyte loss were observed as anticipated since the mechanism of CID-induced apoptosis is independent of glutamate signaling. By contrast, while myelin integrity was disrupted following CID delivery (Figures 7A,C) it was substantially less affected following co-delivery of CID and NMDA receptor antagonists (Figures 7B,D). For example, greater than 80% of myelin sheaths had at least moderate loss of compaction following astrocyte ablation with CID alone (Figures 7E,E’), whereas the addition of the NMDA receptor antagonist QNZ46 reduced the proportion of moderately affected sheaths to 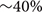 (Figures 7E,E’). Myelin sheaths surrounding large diameter axons were more severely affected by than those associated with small sized axons and the protection by NMDA receptor inhibition was more evident in small and medium sized axons (Figures 7D’,E). Consistent with previous studies in models of ischemia, QNZ46 was more effective than MK801 at myelin sheath protection, possibly as a result of better white matter penetration and myelin sheath selectivity (Doyle et al., 2018).

### Local Delivery of Glutamate to White Matter Results in Reduction in MBP Immunoreactivity and Loss of Myelin Compaction

**FIGURE 8:**
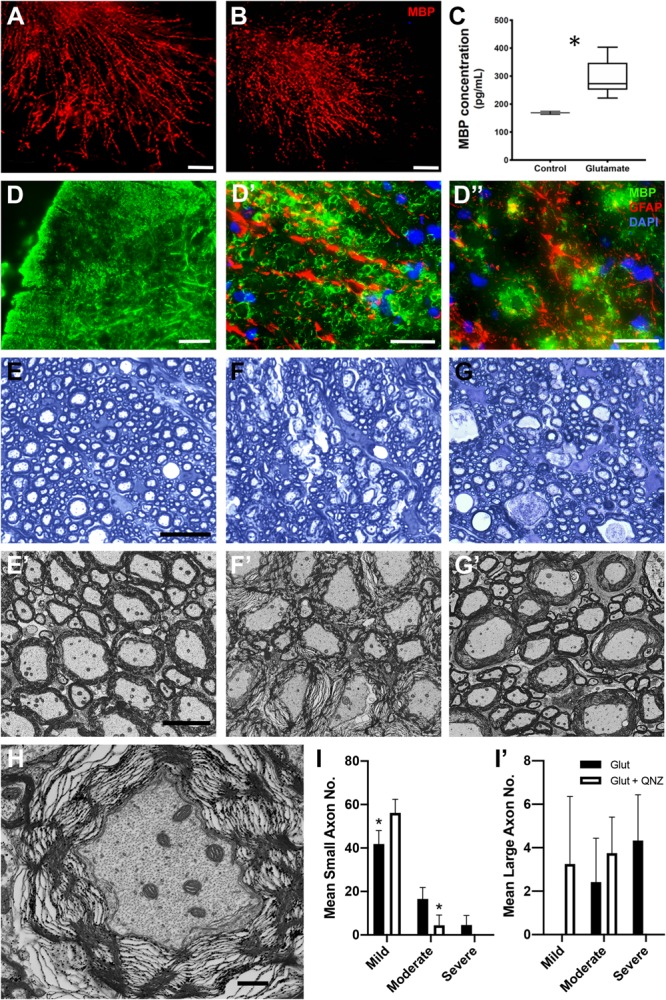
Local elevation of glutamate in WT CNS results in local loss of myelin integrity in the presence of astrocytes. **(A–C)** Treatment of cerebellar slices with glutamate results in a disruption of myelin organization and a release of MBP (red). Control slices in the absence of glutamate **(A)** compared to parallel slices **(B)** treated with 100 μM L-glutamic acid for 24 h. Note the reduction and fragmentation of the MBP processes in CID treated slices compared to controls. Quantitation of MBP by ELISA **(C)** in the conditioned medium of control and (12 h) glutamate treated slices. MBP levels are significantly increased following glutamate treatment. **(D–J)** Injection of glutamate into spinal cord white matter results in a loss of myelin integrity (MBP, green) but not astrocytes (GFAP, red). There is a loss of MBP staining in the region of glutamate injection **(D)** without concomitant loss of astrocytes **(D’)**. **(D”)** Astrocyte processes are retained in regions of MBP loss. (**(E)** Toluidine blue light **(E)** and EM **(E’)** images from spinal cord white matter tracks adjacent to the location of glutamate injection into ventral white matter. Note the preservation of myelin integrity. **(F)** Light **(F)** and EM **(F’)** images taken from white matter tracks in the region of glutamate injection. Note the significant disruption of myelin integrity. **(G)** Light **(G)** and EM **(G’)** images taken from the region of glutamate injection in animals that received co-injection of QNZ46. Note the relative preservation of the myelin sheaths. **(H)** Axonal morphology is relatively unaffected in axons ensheathed by disrupted myelin. **(I)** Quantitation of the relative levels of perturbed myelin sheaths in the presence of glutamate and glutamate + QNZ46 from axons of different diameters: Small **(I)**, and large **(I’)**. Graphs represent mean axon number ± SD. Two-way ANOVA with Sidak’s multiple comparison test. * indicates *p* < 0.001. Scale bars: **(A,B)** 50 μm; **(D)** 100 μm, **(D’,D”)** 25 μm; **(E–G)** 25 μm; **(H–J)** 5 μm; **(K)** 1μm.)

To directly examine the effects of elevated glutamate on myelin integrity, cerebellar slices were exposed to exogenous glutamate (100 μM) for 24 h. A disruption of myelin sheath organization denoted by fragmentation of MBP immunostaining was seen in glutamate treated samples but not in vehicle-treated controls (Figures 8A,B). The fragmentation of MBP processes in glutamate treated slices was correlated with an elevation in detectable MBP protein in the conditioned media from glutamate treated slices (Figure 8C; student *t*-test; *p* < 0.05). To assess the effects of exogenously administered glutamate *in vivo*, adult WT animals were injected, at the T11/13 level of the ventral white matter of the spinal cord, with 2 μl of 100 μM L-glutamic acid, and myelin compaction assayed 24 h later by immunohistochemistry and electron microscopy. In the vicinity of the injection site and in associated axon tracts, several microns rostral and caudal to the point of injection, reduced MBP expression and disruption of GFAP+ astrocytes were apparent (Figures 8D–G). In general, large diameter axons were more severely affected than medium and small diameter ones. Quantitation indicated that greater than 50% of large and 20% of small and medium diameter myelinated axons were affected (Figures 8I,I’). Morphologically, the axons appeared relatively unaffected with intact axolemma and normal distribution of microtubules and neurofilaments (Figure 8H). The glutamate-induced disruption of myelin integrity was largely blocked by co-injection of the NMDA receptor antagonist QNZ46 (Figures 8G,G’). As with CID injections into the GFAP-iCP9 spinal cord the protection of small and medium sized sheaths was more complete than that of large diameter axons suggesting that myelin sheaths around large diameter axons are more susceptible to and more challenging to protect from, glutamate-induced damage (Figures 8I,I’).

## Discussion

The roles of astrocytes in the formation and maintenance of CNS myelin compaction are poorly defined. Using a novel transgenic approach in which an inducible caspase 9 construct is driven by the mouse GFAP promoter we are able to target greater than 85% of GFAP+ cells in the developing and adult CNS. Activation of the iCP9 construct through the local delivery of a small molecule dimerizer (CID) results in the local selective induction of GFAP+ astrocyte apoptosis. Injection of CID into the midline of the corpus callosum in adult GFAP-iCP9 mice induced targeted apoptosis of GFAP+ cells as well as a reduction in the numbers of olig2+ cells. The reduction in oligodendrocyte lineage cells appears to reflect loss of astrocyte derived survival factors since treatment of oligodendrocyte enriched GFAP-iCP9 CNS cultures with CID had no effect on the number of oligodendrocyte lineage cells in the presence of added growth factors. In the spinal cord, the transient induction of astrocyte apoptosis through localized injections of CID during the first postnatal week resulted in delayed myelination that subsequently recovered by postnatal day 15. Extending astrocyte loss into the second postnatal week resulted in a reduction in myelin formation and a profound lack of myelin compaction in the affected regions. Myelination of adjacent (non-CID injected) regions of the CNS and adjacent Schwann cell myelinated PNS were unaffected by CID treatment. In the adult spinal cord, CID induced activation of GFAP+ astrocyte apoptosis resulted in the rapid loss of myelin compaction that was reduced by treatment with NMDA receptor antagonists suggesting it was mediated by local increases in glutamate. Consistent with this hypothesis local injection of glutamate into the adult spinal cord resulted in loss of myelin compaction that was also diminished by co-delivery of NMDA receptor antagonists. Together these studies suggest GFAP+ astrocytes are critical for CNS myelin compaction and maintenance in normal spinal cord white matter.

Previous studies have strongly implicated astrocytes in oligodendrocyte development and myelination (Barnett and Linington, 2013; Lundgaard et al., 2013; Domingues et al., 2016; Molina-Gonzalez and Miron, 2019). For example, OPCs that arise in specific regions of the CNS germinal zone (Pringle and Richardson, 1993; Orentas and Miller, 1996) migrate throughout the CNS (Kessaris et al., 2006), utilizing astrocyte-derived matrix substrates, growth factors (Barnett and Linington, 2013) and localization cues (Tsai et al., 2002; Fancy et al., 2009) before undergoing further proliferation and differentiation. Astrocytes release multiple growth factors including platelet-derived growth factor (PDGF) (Raff et al., 1988), brain-derived neurotrophic factor (BDNF) (Jean et al., 2008), and ciliary neurotrophic factor (CNTF) (Jean et al., 2008; Nash et al., 2011) that promote OPC proliferation and survival (Noble and Murray, 1984; Calver et al., 1998; Fruttiger et al., 1999). Astrocyte-oligodendrocyte coupling via gap junctions also contributes to the maintenance of myelin (Tress et al., 2012), and transgenic loss of connexins 43 and 30 in astrocytes is associated with myelin decompaction and hypomyelination (Lutz et al., 2009). This glial “syncitium” acts as an effective potassium buffer during neurotransmission (Menichella et al., 2006), and provides metabolic support to oligodendrocytes by allowing unidirectional transport of cytosolic contents from astrocytes to oligodendrocytes (Robinson et al., 1993). Astrocytes can also negatively regulate oligodendrocyte development through release of factors such as bone morphogenetic proteins (BMPs) that inhibit the differentiation of OPCs (Hall and Miller, 2004; See and Grinspan, 2009). Astrocytes have also been proposed to act as conduits for sensing axonal activity (Ishibashi et al., 2006), which in turn can stimulate myelination (Barres et al., 1993), while anatomical studies demonstrated that astrocytes contribute to the structure of nodes of Ranvier (Ffrench-Constant et al., 1986), and may influence myelin structure (Dutta et al., 2018). This suggests that astrocytes have multiple roles during the development of CNS white matter.

Here we provide *in vivo* evidence that astrocytes are essential for timely spinal cord myelination. Ablation of GFAP+ astrocytes for 3 days during the first postnatal week markedly reduced the number of CC1+ oligodendrocytes and delayed but not irreversibly blocked myelination suggesting there is a relatively large time window for successful myelination *in vivo*. These findings support previous reports where conditional ablation of dividing GFAP+ astrocytes expressing the herpes thymidine kinase (TK) gene in the first postnatal week seemed to significantly reduce myelination and cause deficits in cerebellar development (Delaney et al., 1996). The reduction of OPCs and oligodendrocytes and lack of myelination in areas of astrocyte ablation likely reflects the loss of growth and survival factors such as PDGF which is consistent with the known expression of PDGFα receptors on OPCs (Pringle and Richardson, 1993). Other mechanisms may also contribute to the delay in myelination in regions of astrocyte ablation. For example, astrocytes contribute to CNS vascularization and blood brain barrier (BBB) formation (Janzer and Raff, 1987) and local defects in BBB function following regional astrocyte ablation may induce a non-conducive environment for OPC survival and myelination. Indeed, disruption of the CNS vasculature is known to affect oligodendrogenesis (Tsai et al., 2016). In addition, if astrocytes are critical in relaying axonal activity to pre-myelinating cells (Ishibashi et al., 2006) then their loss would also lead to the perturbation of myelin formation. The relative contribution of these different mechanisms is currently unclear.

Extending the window of astrocyte ablation to 2 weeks postnatally by injecting CID on alternate days continues to suppress myelination in the spinal cord resulting in hypomyelination and overall loss of myelin compaction. The number of CC1+ positive cells was also reduced but less completely than with the short-term ablation. Compared to short term ablation where oligodendrocytes and myelin were absent, the presence of oligodendrocytes and the formation of non-compact myelin may reflect either the survival of a small cohort of GFAP+ cells and/or the replacement of GFAP+ astrocyte functions by a subset of non-GFAP+ astrocytes that are sufficient to support the development of oligodendrocytes, but are not capable of supporting myelin compaction. Although myelin was decompacted following long-term GFAP+ astrocyte depletion, there was little evidence of peripheral immune cell infiltration into the spinal cord suggesting the myelin disruption resulted directly from the perturbation of astrocyte-based functions. This is consistent with previous reports where GFAP null mice had abnormal myelination but no obvious signs of inflammation (Liedtke et al., 1996), as well as a recent report where diphtheria toxin A mediated astrocyte depletion in adult mice resulted in neuronal loss without evidence of BBB disruption or leukocyte extravasation (Schreiner et al., 2015). Consistent with a central role for astrocyte dysfunction in myelin loss, in an animal model of the demyelinating leukodystrophy vanishing white matter disease (Dooves et al., 2016) the pathology was suggested to reflect mutations in the eukaryotic translation initiation factors EIF2B1-EIF2B5 (Leegwater et al., 2001; Van Der Knaap et al., 2002) in astrocytes. The similarity in pathology and dependence on astrocyte derived factors in this model compared with the current data strongly supports the notion that maintenance of normal white matter is dependent on the normal astrocyte functions.

Several lines of evidence support the hypothesis that local increases in glutamate contribute to the loss of myelin compaction following adult astrocyte ablation. First, the degree of myelin perturbation is substantially reduced by the concomitant delivery of NMDA receptor blockers such as QNZ46. Second, direct delivery of glutamate to adult spinal cord white matter results in rapid loss of myelin compaction that mimics the pathology seen following astrocyte ablation, and is in line with earlier reports showing that over-activation of glutamate receptors in the acute setting in rodents produces well-circumscribed areas of demyelination reminiscent of MS plaques (Matute et al., 1997, 2001). The notion that GFAP+ astrocyte ablation results in elevated extracellular levels of glutamate supports earlier work showing increased extracellular glutamate after inducible ablation of astrocytes (Cui et al., 2001), or following selective knockdown of their glutamate transporters (Rothstein et al., 1996).

It seems likely that the glutamate released as a result of astrocyte ablation binds to GluN2C/D-containing NMDA receptors on oligodendrocytes or myelin leading to the rapid loss of myelin, and is at-least partially reversable by use of the selective NMDA receptor antagonist QNZ-46 (Karadottir et al., 2005; Salter and Fern, 2005; Micu et al., 2006; Doyle et al., 2018). It is possible that some of the effects on myelin are secondary to glutamate-induced axonal damage, however, contrary to previous reports (Delaney et al., 1996; Schreiner et al., 2015) we did not see any ultrastructural evidence of axonal disruption, although this may develop subsequently. NMDA receptors have been described on oligodendrocytes and deletion of the canonical GluN1 (NR1) subunit of NMDA receptors from oligodendrocytes but not their progenitors results in delayed myelination and a reduced capacity of oligodendrocytes to provide trophic support to their target axons (Guo et al., 2012; Saab et al., 2016). In the setting of ischemic insults it has been proposed that glutamate derived from axonal vesicles results in local perturbation of myelin structure mediated through GluN2C/D subunits that are selectively inhibited by QNZ46 (Doyle et al., 2018). The observation that following astrocyte loss QNZ46 is effective in protecting myelin integrity is consistent with these recent findings. Since activation of NMDA receptors promotes rapid loss of myelin compaction it is surprising that deletion of these receptors from oligodendrocytes does not alter the progression of EAE, an immune mediated demyelinating model (Guo et al., 2012). This apparent lack of effect implies that in EAE other mechanisms of white matter damage such as direct damage by T cells or inflammation dominate over glutamate-induced myelin disruption.

Mechanisms other than a direct effect of elevated glutamate on oligodendrocyte or myelin may also contribute to the decompaction of myelin seen following GFAP+ astrocyte loss. For example, the numbers of CC1+ oligodendrocytes were markedly reduced in areas of GFAP+ astrocyte loss supporting the concept that *in vivo* oligodendrocyte survival is dependent on astrocytes. Oligodendrocyte loss or dysfunction has been at the center of pathological events described in demyelinating diseases such as MS, and *in vivo* manipulation of oligodendrocyte function almost invariably results in myelin defects (Domingues et al., 2016). Astrocytes are implicated in providing energy sources to oligodendrocytes (Rinholm et al., 2011), and if maintaining myelin compaction were a highly energy dependent process, then loss of astrocytes would result in decompaction by affecting oligodendrocyte function. It seems unlikely, however, that myelin decompaction simply reflects oligodendrocyte death since following induction of oligodendrocyte death by iCP9 induction (Pajoohesh-Ganji and Miller, 2019) or experimentally induced DTA-mediated cell depletion myelin remained relatively intact for a considerable time following oligodendrocyte death (Traka et al., 2010, 2016) that likely reflects the slow turnover of myelin components in the adult brain (Norton and Poduslo, 1973; Benjamins and Morell, 1978). In the setting of advanced disease however, it seems likely that multiple mechanisms will contribute to myelin pathology.

The observation that, in the mature CNS, local astrocyte ablation results in myelin decompaction and demyelination raises the possibility that astrocytes are an important target in demyelinating diseases. In NMO the optic nerve and spinal cord are selectively targeted with immune cell infiltration, astrocyte loss, and demyelination (Sellner et al., 2010). A proportion of NMO patients are seropositive to aquaporin 4 (AQP4) antibody (Lennon et al., 2004, 2005), a membrane bound water channel that is preferentially expressed at the end feet of astrocytes (Amiry-Moghaddam et al., 2003). Binding of AQP4 antibodies (anti-AQP4 IgG) is thought to result in complement mediated astrocyte cytotoxicity or loss of function, that results in macrophage infiltration, oligodendrocyte loss and demyelination (Marignier et al., 2010; Wrzos et al., 2014). However, it has been reported that in NMO, anti-AQP4 IgG reduces glutamate uptake by astrocytes through down-regulation of the expression of excitatory amino acid transporter 2 (EAAT2), which is responsible for most of the glutamate uptake in the CNS (Hinson et al., 2008), raising the possibility that perturbation of glutamate homeostasis may contribute to myelin pathology in NMO (Marignier et al., 2010).

## Conclusion

In conclusion, here we show that loss of GFAP+ astrocyte compromises myelination during development and results in rapid myelin decompaction and demyelination in adult white matter. These studies provide strong evidence that astrocytes may represent a potential cellular target in a range of demyelinating diseases.

## Data Availability Statement

The datasets generated for this study are available on request to the corresponding author.

## Ethics Statement

The animal study was reviewed and approved by the Institutional Animal Care and Use Committees at Case Western Reserve University and The George Washington University School of Medicine and Health Sciences.

## Author Contributions

RT conceived the study, performed experiments, analyzed the data, and drafted the manuscript for intellectual content. MK and MA-R performed experiments, analyzed the data, and drafted the manuscript for intellectual content. SF-M, AP, EG, and JS assisted with experimental design and completion. RM conceived the study, analyzed the data, and drafted the manuscript for intellectual content.

## Conflict of Interest

SF-M was employed by the company Ultragenyx. The remaining authors declare that the research was conducted in the absence of any commercial or financial relationships that could be construed as a potential conflict of interest.
